# Identification of a novel *UMOD* mutation (c.163G>A) in a Brazilian family with autosomal dominant tubulointerstitial kidney disease

**DOI:** 10.1590/1414-431X20176560

**Published:** 2018-03-01

**Authors:** L.B. Lopes, C.C. Abreu, C.F. Souza, L.E.R. Guimaraes, A.A. Silva, F. Aguiar-Alves, K.O. Kidd, S. Kmoch, A.J. Bleyer, J.R. Almeida

**Affiliations:** 1 Laboratório Multiusuário de Apoio è Pesquisa em Nefrologia e Ciências Médicas (LAMAP), Departamento de Medicina Clínica, Faculdade de Medicina, Universidade Federal Fluminense, Niterói, RJ, Brasil; 2 Laboratório Multiusuário de Apoio è Pesquisa em Nefrologia e Ciências Médicas (LAMAP), Departamento de Patologia, Faculdade de Medicina, Universidade Federal Fluminense, Niterói, RJ, Brasil; 3 Programa de Pós-Graduação em Patologia, Faculdade de Medicina e Laboratório Rodolpho Albino, Universidade Federal Fluminense, Niterói, RJ, Brasil; 4 Departamento de Ciências Básicas, Polo Universitário de Nova Friburgo, Universidade Federal Fluminense, Nova Friburgo, RJ, Brasil; 5 Section on Nephrology, Wake Forest School of Medicine, Winston-Salem, NC, USA; 6 Institute for Inherited Metabolic Disorders, and First Faculty of Medicine, Charles University in Prague, Prague, Czech Republic

**Keywords:** Chronic kidney disease, Uromodulin kidney disease, Genetic mutation, Uromodulin, UMOD, Genetic kidney diseases

## Abstract

Autosomal dominant tubulointerstitial kidney disease (ADTKD) is characterized by autosomal dominant inheritance, progressive chronic kidney disease, and a bland urinary sediment. ADTKD is most commonly caused by mutations in the *UMOD* gene encoding uromodulin (ADTKD-*UMOD*). We herein report the first confirmed case of a multi-generational Brazilian family with ADTKD-*UMOD*, caused by a novel heterozygous mutation (c.163G>A, GGC→AGC, p.Gly55Ser) in the *UMOD* gene. Of 41 family members, 22 underwent genetic analysis, with 11 individuals found to have this mutation. Three affected individuals underwent hemodialysis, one peritoneal dialysis, and one patient received a kidney transplant from a family member later found to be genetically affected. Several younger individuals affected with the mutation were also identified. Clinical characteristics included a bland urinary sediment in all tested individuals and a kidney biopsy in one individual showing tubulointerstitial fibrosis. Unlike most other reported families with ADTKD-*UMOD*, neither gout nor hyperuricemia was found in affected individuals. In summary, we report a novel *UMOD* mutation in a Brazilian family with 11 affected members, and we discuss the importance of performing genetic testing in families with inherited kidney disease of unknown cause.

## Introduction

Autosomal dominant tubulointerstitial kidney disease is characterized by a bland urinary sediment and progressive chronic kidney disease. Multiple names have been proposed for this group of disorders, including medullary cystic kidney disease, familial juvenile hyperuricemic nephropathy, and uromodulin associated kidney disease. A recent consensus from a conference for improving global outcomes in kidney disease (KDIGO) (1) proposed a new nomenclature for this group of diseases, using the term autosomal dominant tubulointerstitial kidney disease (ADTKD) as a collective term, with further nomenclature determined by the underlying genetic mutation. ADTKD-*UMOD* is caused by mutations in the *UMOD* gene encoding uromodulin. In addition to kidney disease, ADTKD-*UMOD* families often suffer from gout and hyperuricemia. ADTKD-*REN* is caused by mutations in the *REN* gene encoding renin and is associated with childhood anemia, hyperuricemia, gout, and hyperkalemia (2). ADTKD-*MUC1* is caused by a frameshift mutation in the *MUC1* gene; there are no other clinical findings except for progressive chronic kidney disease (3). ADTKD is also caused by mutations in the *TCF2* gene encoding hepatocyte nuclear factor 1 beta (4). In this condition, early onset diabetes, abnormalities in liver function tests, and congenital abnormalities of the genito-urinary tract may occur. Recently, mutations in the *SEC61A1* gene were also identified as a cause of ADTKD (5). Patients with this condition may have anemia, developmental delay, and/or leukopenia. Of all the subtypes of ADTKD, ADTKD-*UMOD* is the most common (6,7).

Uromodulin, also known as Tamm-Horsfall protein, is encoded by the *UMOD* gene on chromosome 16p12 (8). It is produced exclusively by tubular cells in the thick ascending limb of the loop of Henle and is the most common protein excreted in the urine (9). While its function is not fully understood, it has been found to facilitate transport of the furosemide-sensitive NKCC2 transporter to the apical surface of the thick ascending limb (10). As a result, there is defective sodium transport in the thick ascending limb, resulting in a mild natriuresis. This natriuresis leads to secondary proximal tubular sodium uptake as well as urate uptake, resulting in hypouricosuric hyperuricemia and gout. Mutations in the *UMOD* gene also result in a misfolding of the uromodulin protein, resulting in deposition of the mutant uromodulin in the endoplasmic reticulum (11). Some families with *UMOD* mutations have a milder clinical course, without hyperuricemia and gout and milder manifestations of chronic kidney disease (12,13 [Bibr B14]).

The present study describes a new *UMOD* mutation for the first time in a Brazilian family presenting with ADTKD but without gout or hyperuricemia.

## Patients and Methods

**Figure 1.**
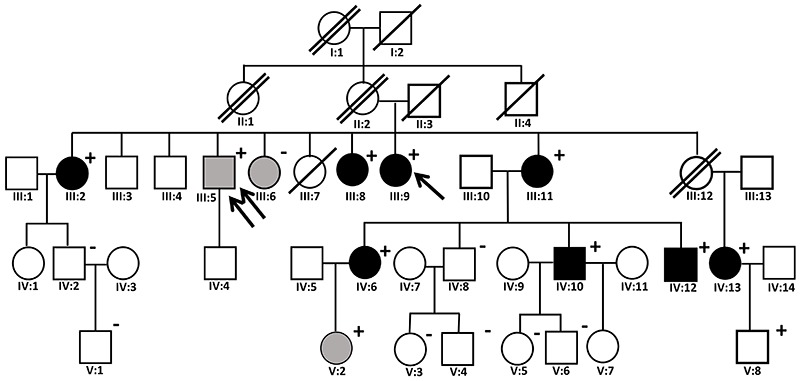
Family pedigree. The arrow indicates the index case. The double arrow indicates the patient who donated a kidney and still had an estimated glomerular filtration rate (eGFR) of 66 mL·min^-1^/(1.73m^2^) at age 59. One transverse bar means death by unknown cause whereas two transverse bars mean death in hemodialysis. Black figures indicate end stage renal disease or eGFR <60 mL·min^-1^/(1.73m^2^). Gray figures indicate eGFR >60 and <90 mL·min^-1^/(1.73m^2^). For patients affected clinically, see Supplementary Table S1 for details. A plus sign (+) indicates that the patient was genetically tested and found to have the *UMOD* mutation. A negative sign (−) means the patient was genetically tested and found not to have a *UMOD* mutation.

The index case was a 62-year old woman who had suffered from chronic kidney failure of unknown cause for the preceding 5 years. Laboratory studies revealed a serum creatinine of 2.19 mg/dL [CKD-EPI estimated glomerular filtration rate (eGFR)=24 mL·min^-1^/(1.73m^2^)]. The urinalysis revealed no hematuria, with a spot urinary protein:creatinine ratio of 290 mg/g. The serum urate level was 5.0 mg/dL. There was a strong family history of kidney disease, with many affected family members (see Figure 1).

After a syndromic diagnosis of ADTKD was made, we conducted a genetic study in all family members who were willing to participate. A written consent term was created and approved by the Ethical Committee of the Hospital Universitário Antonio Pedro, Universidade Federal Fluminense. Blood samples were obtained, and the DNA was extracted from whole blood using a commercial QIAamp DNA kit (QIAGEN, Germany) following the manufacturer's instructions. Exon and intron 4 and exon 5 of the *UMOD* gene were amplified using the following *UMOD* X4-F primers (5′-GGTGGAGGCTTGACATCATCAGAG-3′) and *UMOD* X5-R (5′-GGAATAGGGCTCAGATGGTCTTTG-3′), as previously described (see Ref. 14). Polymerase chain reaction (PCR) was performed on a thermocycler (Veriti® 96-Well Thermal Cycler model, Applied Biosystems, USA) under the following conditions: a denaturation cycle at 95°C for 5 min, hybridization in 35 cycles of 95°C for 30 s, 56°C for 30 s, and 68°C for 90 s and extension at 68°C for 10 min. The reaction product was purified using the Wizard SV gel kit (Promega, USA). Sequencing was performed with the BigDye® Terminator Cycle Sequencing V3.1 kit (Applied Biosystems™, USA) and analyzed on the 3130 Genetic Analyzer Sequencer (Applied Biosystems, USA). Sequencing of the *REN* gene was performed at the First Faculty of Medicine, Charles University, Prague, Czech Republic, and genetic testing for ADTKD-*MUC1* was performed at the Broad Institute of Harvard Medical School and the Massachusetts Institute of Technology, Cambridge, MA, USA (15).

## Results

**Figure 2.**
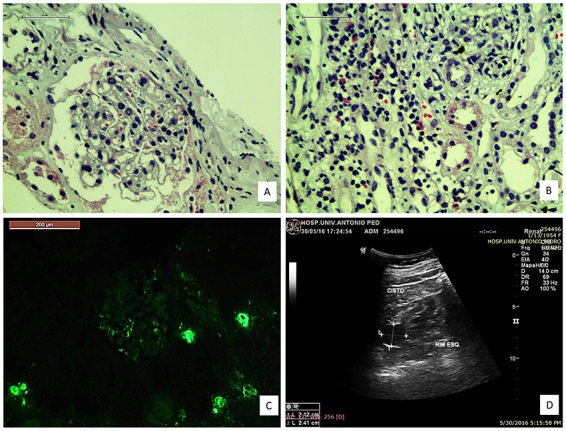
Renal biopsy photomicrography and ultrasound images. *A*, Normal glomerulus (hematoxylin-eosin; bar=50 µm). *B*, Tubulointerstitial area; mild interstitial fibrosis and tubular atrophy with mononuclear and eosinophilic inflammatory infiltrate (hematoxylin-eosin, bar=50 µm). *C*, Negative immunostaining of the glomerulus for immunoglobulin G. (*A*, *B* and *C* from case IV:6). *D*, Renal ultrasound image showing hyperechogenic kidney with cortical cyst from case III:9.

The renal ultrasound of the index case revealed small bilateral hyperechogenic kidneys, with some small corticomedullary cysts (Figure 2D). All usual serologic markers including complement proteins, anti-nuclear antibodies, as well as viral serologies were negative. It was decided to clinically characterize all family members, including a complete medical history, physical examination, laboratory testing, and renal ultrasound. From this data, we determined that the condition was inherited in an autosomal dominant manner.

Most of the affected individuals of the family presented with an unexplained decline in glomerular filtration rate. Recurrent urinary tract infections were identified in several female members. There was no significant proteinuria, hematuria, gout or hyperuricemia. Juvenile diabetes was not present, and blood pressure readings were normal in all individuals. Individual biochemistry data, when available, are reported in Supplementary Table S1. A kidney biopsy performed in one affected family member revealed tubular atrophy, interstitial inflammation, and negative immunostaining for immunoglobulins and complement proteins (Figure 2A–C). In several patients, renal ultrasounds revealed a hyperechogenic cortex and occasional small corticomedullary cysts. Taken together, the clinical presentation of this family was considered characteristic of ADTKD.

**Figure 3.**
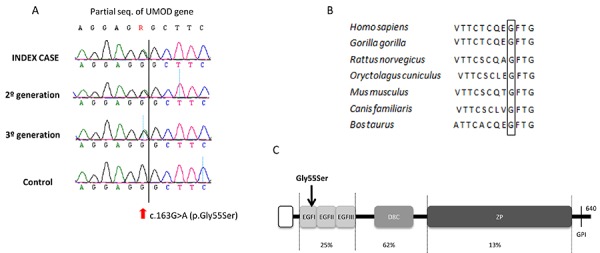
Sequence analysis, evolutionary conservation and uromodulin protein diagram. *A*, *UMOD* gene analysis at exon 4 from the index case and two family members from other generations. The sequence analysis showed a novel heterozygous missense mutation, G163A, resulting in a G55S amino acid exchange. The arrow indicates the overlapping peaks at the site of nucleotide substitution. All positive patients presented the same mutation. The cases are III:9, IV:6 and V:2, respectively. *B*, Conservation of the mutated amino acid G55 residue (boxed) among mammals. *C*, Structure of the uromodulin protein, showing a leader peptide, three epidermal growth factor EGF-like domains, a central domain named D8C, a ZP domain and a glycosylphosphatidylinositol GPI-anchoring site; the black arrow shows the heterozygous missense mutation c.G163A; p.Gly55Ser, found in the present study, located in EGF1 domain. Below each region are the percentages of mutations reported to date.

Genetic analysis was performed on 22 individuals, including 3 non-consanguineous spouses who served as controls. A mutation was not identified in the *REN* or *MUC1* gene. Of the 22 participants, 11 individuals were found to have a novel c.163G>A, GGC→AGC, p.Gly55Ser mutation in exon 4 of the *UMOD* gene (Figure 3A). The mutation occurred in exon 4, a frequent site for *UMOD* mutations (16–18). Figure 1 shows the family pedigree. Figure 3A and Supplementary Table S1 show genotype and phenotype details of the family members.

The conservation of the mutated amino acid residues was assessed by BlastP alignment of human wild-type protein and other mammalian uromodulin homologs (*Gorilla gorilla*, *Rattus norvegicus*, *Oryctolagus cuniculus*, *Mus musculus*, *Canis familiaris* and *Bos taurus*) (Figure 3B), with the wild-type amino acid being conserved across all species. A diagram was created showing the domains of the uromodulin protein and the position of the identified mutation (Figure 3C). In order to predict the functional effects of the missense mutation, we used the Polyphen 2 program (Polymorphism Phenotyping v2, http://genetics.bwh.harvard.edu/pph2/), the Sorting Intolerant From Tolerant program (SIFT, http://sift.jcvi.org/), and the M-CAP score (13). The PolyPhen2 core predicted a 40% likelihood of pathogenicity. The SIFT program predicted a 53% likelihood of pathogenicity. The M-CAP score for p.G55S is 0.617, indicating the mutation is "possibly pathogenic". The LOD (logarithm of odds) score was calculated as 2.58, with a recombination frequency of 0.15. Genetic testing of the *MUC1* and *REN* genes was negative. Based on the clinical presentation, the presence of the mutation in all affected individuals, the presence of the mutation in a hotspot for *UMOD* mutations, and the negative results from other genetic analysis, the family was diagnosed with ADTKD-*UMOD*.

After genotyping, further clinical analysis of genetically affected individuals could be performed. Of 10 affected individuals in whom laboratory data was available, 6 had advanced chronic kidney disease [eGFR <60 mL·min^-1^/(1.73m^2^)], undergone kidney transplantation or started dialysis. One child (V:8) had normal renal function and one 28-year-old (V:2) had mildly decreased renal function [eGFR 89 mL·min^-1^/(1.73m^2^)]. There was also a 59-year-old genetically affected male (III:5) who had donated a kidney prior to genetic testing; his eGFR was 66 mL·min^-1^/(1.73m^2^).

## Discussion

Several previous studies have revealed various heterozygous missense mutations in the *UMOD* gene of families with ADTKD. The renal clinical phenotype caused by *UMOD* mutation is characterized by dominant inheritance, chronic kidney disease due to chronic tubulointerstitial nephritis, hyperuricemia, gout, and, occasionally, renal cysts (19–21). More than 100 mutations in the *UMOD* gene have been described; the majority of reported *UMOD* mutations cluster in exons 4 (>80%) and 5 (>11%), resulting in the replacement of cysteine residues and leading to misfolding of the uromodulin molecule (22,23).

In the family under study, serum urate levels were normal, and a number of family members had relatively well-preserved kidney function. Increased awareness of ADTKD-*UMOD* as a cause of inherited kidney disease, together with increased genetic testing, has revealed a variation in the severity of chronic kidney disease and the presence of gout and hyperuricemia between families. In one recent study of 202 patients from 74 families, 91% of tested patients had hyperuricemia (24). However, in a study of a family with an in-frame *UMOD* indel (insertion and deletion) mutation, only 1/30 family members had gout and hyperuricemia (12). Thus, gout is not found in all families with ADKTD-*UMOD*. In the current family, there was a wide variation in age of onset of end-stage kidney disease, with patients starting dialysis at 44, 56, and 60 years, while two family members had well preserved kidney function at ages 59 and 62. The 62-year-old patient had actually donated a kidney to a family member and still retained kidney function later in life. The wide variation of age of ESRD has been noted by other investigators, with some affected family members starting dialysis in their 30's while others had not started dialysis into their late 70's (25). The cause of this variation in age of onset of ESRD is unknown. Recently, Bleyer et al. have identified seven cases of genetically affected individuals with ADTKD donating a kidney and retaining surprisingly good kidney function over time (Bleyer AJ, et al. Abstract, American Society of Nephrology Kidney Week, 2017). The reason for milder disease in some individuals remains unexplained. However, researchers are trying to identify the factors responsible for improved kidney function in some individuals to see if they could point to potential therapies.

ADTKD-*UMOD* is a rare disease. The true prevalence is difficult to determine because the condition is frequently underdiagnosed (26). The findings of slowly progressive renal failure, bland urinalyses, and unremarkable renal ultrasounds make the correct diagnosis elusive. Families with ADTKD-*UMOD* have been reported from Europe, USA, Asia, and Africa (6). A nationwide epidemiologic survey of ADTKD-*UMOD* conducted in Austria revealed a prevalence of 1.7 cases per million population and 1 case per 1000 renal replacement therapy patients (27).

Medeiros et al. (28) reported a Brazilian family with history of juvenile gout and chronic kidney disease and made a clinical diagnosis of familial juvenile hyperuricemic nephropathy; a mutational analysis of the *UMOD* gene was not performed.

At present, there is no specific therapy for ADTKD-*UMOD*. Allopurinol is very effective as a treatment of hyperuricemia and gout in these patients, but it is unclear if it has an effect on progression of chronic kidney disease (29–31). Even though there is no specific therapy for ADTKD-*UMOD*, there are several reasons to perform genetic testing. First, definitive diagnosis is very important for many family members and their doctors. A correct diagnosis will prevent unnecessary testing and avoid unneeded kidney biopsies. Second, genetic testing can then be performed to confirm the presence or absence of disease in other family members. Genetic counselling can also be provided. Correct identification will also allow a better characterization of this condition over time. Finally, when a treatment does become available, family members will have already been identified and can be notified. Even though there was a successful donation in a genetically affected donor, we still believe that all family members who are potential donors should be tested for the familial mutation and very great caution be exercised when considering them for donation.

In summary, we have identified a multi-generational family in Brazil in which a mutation in the *UMOD* gene is linked to ADTKD. The family had a less severe presentation compared to other families, with several family members being mildly affected, and with the absence of hyperuricemia and gout. Nephrologists should consider *UMOD* mutational analysis in families with ADTKD and a bland urinary sediment, even when hyperuricemia and gout are not present.

## Supplementary Material

Click here to view [pdf]
